# Intermittent Explosive Disorder in Male Juvenile Delinquents in China

**DOI:** 10.3389/fpsyt.2019.00485

**Published:** 2019-07-26

**Authors:** Yang Shao, Yi Qiao, Bin Xie, Min Zhou

**Affiliations:** Department of Forensic Psychiatry, Shanghai Mental Health Center, Shanghai Jiao Tong University School of Medicine, Shanghai, China

**Keywords:** intermittent explosive disorder, juvenile delinquent, aggression, anger, China

## Abstract

**Objective:** Although several previous studies have focused on the mental health problems in detained juvenile offenders in China and found high levels of major psychiatric morbidity, the prevalence of intermittent explosive disorder (IED) in this group remains unknown. The purpose of this study is to discover the prevalence of IED among juvenile offenders in China as well as the difference in demographic characteristics and personality traits between IED offenders and the general population.

**Method:** A total of 280 delinquent boys (Mean age 16.10 years) were interviewed by trained psychiatrists. The interview procedure included the recording of sociodemographic characteristics, criminal records, Composite International Diagnostic Interviews (CIDI), State-Trait Anger and Expression of Anger Inventory-2 (STAXI-2) and Modified Overt Aggression Scales (MOAS).

**Results:** Of the 280 delinquent boys, 32 (11.4%) were diagnosed with IED, 129 (46.1%) were non-IED psychopathology controls (PC), and 119 (42.5%) were healthy controls (HC). Except for substance use disorder (SUD), no differences in psychiatric comorbidity were found between youths with IED and those with another psychiatric disorder. Compared with the PC and HC groups, those in the IED group were more likely to commit a violent crime such as rape, assault, or an affray but it is less likely that their motive can be explained by money or property. The IED group also had a higher rate of recidivism history than the HC group. The IED group displayed higher levels of state and trait anger and anger expression than the HC group and lower levels of anger control than both the PC and HC groups. MOAS also showed that those in the IED group were more aggressive than those in the PC and HC groups.

**Conclusion:** The relationships between IED, anger and aggression reflect the need to develop and implement specific and individually tailored intervention approaches to correct IED juvenile offenders’ behavior in order to prevent new crime.

## Introduction

Intermittent explosive disorder (IED) is a psychiatric condition characterized by recurrent aggressive behavior, explosive outbursts towards people and property, impaired emotion regulation and behavioral control, and it is often comorbid with other psychiatric disorders (1). IED was once considered a relatively rare disorder, but recent studies have reported diverging prevalence estimates. In the United States, while the data provided in the Collaborative Psychiatric Epidemiology Surveys showed that the prevalence of IED was in only 1.62% of the population (2), the National Comorbidity Survey in 2001 to 2003 found that the lifetime prevalence of IED was 7.3% and 5.4% when broad and narrow criteria of IED were used, respectively (3). Further, in the National Comorbidity Survey Replication Adolescent Supplement (NCS-A), researchers found that 7.8% of adolescents between the ages of 13 and 17 met the *Diagnostic and Statistical Manual of Mental Disorders, Fourth Edition* (DSM-IV) criteria for lifetime IED (4). Meanwhile, for studies in other countries, the mean lifetime prevalence of IED was 3% for the broad definition and 1.6% based on the narrow criteria (1).

Due to the high aggression, anger, hostility, and poor temper control characteristics of IED, a series of studies were carried out which focused on the legal consequences of IED. They found a clear linkage between IED and criminal behavior. For example, in one federal probation jurisdiction in Midwestern United States, DeLisi studied 863 federal correction clients’ data and found that those with IED were more likely to be arrested for murder, attempted murder, interference with police, and assault (5). In Chile, Mundt found that the IED prevalence rate was 5.7% among a nationwide random sample of 1,008 prisoners in seven penal institutions (6). Similar associations also existed in the youth population. According to the data from NCS-A (4, 7), when compared to youth without IED, youth with lifetime IED diagnoses had a significantly greater probability of being arrested for burglary or theft (OR = 6.2), violent crime (OR = 11.0), or any other crime (OR = 8.8).

Over the past several decades, juvenile delinquency has emerged as a serious social problem in China (8). As China began its transformation into a market economy in the late 1970s, the problem of juvenile crimes became increasingly troublesome. In 1990, the number of juvenile offenders under the age of 18 adjudicated by the courts was 42,033, with that number rising steadily to 55,817 in 2013, thereby representing an increase of over 33% (9).

Recently, studies focused on the mental health problems of detained juvenile offenders in China have found high levels of major psychiatric morbidity, including attention deficit hyperactivity disorder (ADHD), oppositional defiant disorder (ODD) and conduct disorder (CD) (10). These findings demonstrate that juvenile delinquents in the justice system constitute a population with an elevated risk of psychiatric problems (11), showing that it is critically important to address the psychiatric needs of juvenile offenders (12, 13). One drawback of Zhou’s study was that it did not mention whether or not IED existed in this population. Since research had confirmed that IED had a comparatively high prevalence (1.7%) in metropolitan China (14), we believe that the incidence of IED in juvenile offenders cannot be ignored.

To date, few studies have investigated IED within a criminal justice context in China. In order to account for the gaps in previous literature, the present study sought to identify IED in male juvenile offenders in China. We investigated the anger-related traits and aggressive behavior in individuals with IED, psychiatric controls, and healthy volunteers in a population of juvenile offenders. The differences between the three groups were compared. We hypothesized that youths with IED would have escalated tendencies of anger and aggressive behaviors compared to those in the other two groups. We also predicted that higher anger and aggression in IED would result in different crime profiles than those in the comparison groups.

## Material and Methods

### Participants and Procedure

This cross-sectional study was conducted in the only juvenile reformatory in Shanghai, China. All literate male juvenile delinquents incarcerated in this facility from March to August 2012, were included in the study after obtaining informed consent from both themselves and their parents or guardians.

Participants who agreed to participate were instructed to complete all the questionnaires within 30 days after being sent to the reformatory. All questionnaires that were answered completely and sufficiently were included in the study.

During the 6 months of our study, 347 delinquent boys were incarcerated at this reformatory after being disposed by juvenile courts. Finally, 280 (80.69%) of the boys who responded and completed the questionnaires properly were included in the study.

### Sociodemographic Characteristics and Crime-Related Variables

Through official police records, we collected information regarding age, household registration, most recent offenses (crime type), and criminal history. A self-report questionnaire designed by the authors was used to collect information about the participants’ and their parents’ education levels, as well as other family and living situations. In addition, we also asked participants to report the motives of their crimes in the questionnaire (e.g., money-oriented or otherwise).

### Psychiatric Interview Measures

The Composite International Diagnostic Interview (CIDI), a fully structured interview administered by trained interviewers (15), was conducted to assess the current psychiatric diagnoses of all participants. CIDI has been used in cross-cultural settings with great success (16). DSM‐IV criteria (17) were used to assess the participants’ Axis I Disorders, and IED was diagnosed using research criteria (17, 18). The IED participants also met the new DSM‐5 criteria for IED (19).

### Self-Ratings of Anger

Anger was assessed by the Spielberger State-Trait Anger Expression Inventory-2 (STAXI-2) (20). STAXI-2 is a 57 item self-report scale that is used widely to measure state and trait anger, and it contains several subscales. State anger measures the amount of anger that is experienced at a particular time. Trait anger measures one’s disposition to experience angry feelings. Anger Expression-Out measures the level of verbal and physical anger expressed outwardly at other individuals or objects, while Anger Expression-In measures inward expression of angry feelings (i.e., suppression). Anger Control-Out measures the control of angry feelings by preventing their outward expression, while Anger Control-In measures the level of controlling suppressed anger by calming oneself down. Cronbach alphas for the Chinese version of STAXI-2 were 0.91 for Trait Anger, 0.81 for State Anger, 0.68 for Anger Expression-In, 0.71 for Anger Expression-Out, 0.82 for Anger Control-In, and 0.87 for Anger Control-Out (21).

### Aggression Behavior Measures

Aggressive behaviors of these participants within 30 days (their first month in juvenile reformatory) were measured with the Modified Overt Aggression Scale (MOAS) by one researcher. They were based on the participants’ self-reporting as well as records provided by prison staff. MOAS is a four-part behavior rating scale designed to measure four types of aggressive behavior as witnessed in the past weeks. Each section consists of five questions, with the first section related to verbal aggression, the second focused on aggression against property, the third measuring auto aggression, and the fourth concerning physical aggression (22). After being translated into Chinese, the MOAS has been found to have modest reliability and validity in psychiatric inpatient samples in China (23).

### Ethics

All relevant authorities, including those at the correctional institution and the head of the Shanghai Justice Bureau, were approached. They all granted permission to conduct the assessments and approved our inspection of related files. The Research Ethical Committee at Shanghai Mental Health Center (IORG Number: IORG0002202; FWA Number: FWA00003065) approved the study.

Participants received verbal and written information about the purpose and design of the study. They were informed that their participation was voluntary and that any participant could withdraw without penalty at any time. They were also informed that their responses would remain anonymous, and that only the researchers would see their individual questionnaires.

### Statistical Analysis

The data were analyzed using the software package SPSS 19.0 (SPSS Inc., Chicago, IL). The frequencies and means of the demographic variables of the study were first calculated by descriptive statistics. Data were presented as means ± standard deviations.

The differences between demographic variables, crime profiles, and anger prevalence among the three diagnostic groups were analyzed by ANOVAs and chi-squared tests. Kruskal–Wallis tests were used to compare the differences in severity of the aggressive behaviors between the three groups, as the MOAS scores were ordinal variables. The results of multiple comparisons were presented when there was significant statistical difference within the three groups.

## Results

### Socio-Demographic Characteristics and Crime Profile

Among the 280 participants, 32 (11.4%) participants fulfilled the research criteria for IED (IED group), 129 (46.1%) participants had other DSM-IV psychiatric disorders (psychopathology control group, PC), and 119 (42.5%) participants had no history of psychiatric disorders (healthy control group, HC).

**Table 1** shows the analyses of the demographic variables (age, education, and parental education levels) and the crime profiles. Results showed no group differences with regard to age (all aged 15 to 17 years), educational level (from 4 to 11 completed years), proportion of those who were the family’s only child, household registration (natives of Shanghai vs. migrants from other regions), family income status, parental marital status, and parental education level. Compared with the other two groups, the IED group had a higher rate of violent crimes (mainly rape, assault, and affray) and a lower rate of attributing their motivation to stealing money or property (*P* < 0.001). The rate of recidivism in the IED group was much higher than that of the HC group (*P* < 0.05) and similar to the PC group.

**Table 1:** Socio-demographic characteristics and crime profile of different groups.

	Total *N* = 280	IED *n* = 32	PC *n* = 129	HC *n* = 119	F/χ2	*P*	IED vs. PC	IED vs. HC	PC vs. HC
**Continuous variables**									
Age (year)	16.10 ± 0.61	16.06 ± 0.62	16.11 ± 0.62	16.10 ± 0.62	0.072	0.931	−	−	−
Education (year)	8.20 ± 1.64	8.72 ± 1.49	7.98 ± 1.64	8.29 ± 1.64	2.966	0.053	−	−	−
Father’s education (years)	9.25 ± 3.45	9.88 ± 3.64	9.15 ± 3.38	9.19 ± 3.45	0.607	0.546	−	−	−
Mother’s education (years)	8.34 ± 3.44	9.69 ± 4.10	8.06 ± 3.15	8.29 ± 3.49	2.934	0.055	−	−	−
**Categorical variables**									
The only child in family	192, 68.6%	20, 62.5%	97, 75.2%	75, 63.0%	4.871	0.088	−	−	−
Parental marital status (divorced)	61, 21.8%	5, 15.6%	33, 25.6%	23, 19.3%	2.225	0.329	−	−	−
Family income (monthly)^a^					8.344	0.214	−	−	−
<1,000	29, 10.4%	2, 6.3%	16, 12.4%	11, 9.2%					
1,000–1,999	75, 26.8%	6, 18.8%	39, 30.2%	30, 25.2%					
2,000–3,499	96, 34.3%	17, 53.1%	41, 31.8%	38, 31.9%					
>3,500	80, 28.6%	7, 21.9%	33, 25.6%	40, 33.6%					
Migrant population (*n*, %)	172, 61.5%	16, 50.0%	86, 66.7%	70, 58.8%	3.599	0.165	−	−	−
Types of crime					90.824	<0.001			
Homicide	7, 2.5%	2, 6.3%	2, 1.6%	3, 2.5%			−	−	−
Rape	13, 4.6%	6, 18.8%	6, 4.7%	1, 0.8%			<0.05	<0.05	>0.05
Assault	31, 11.1%	11, 34.4%	15, 11.6%	5, 4.2%			<0.05	<0.05	<0.05
Robbery	50, 17.9%	2, 6.3%	32, 24.8%	16, 13.4%			<0.05	>0.05	<0.05
Affray	32, 11.4%	10, 31.3%	15, 11.6%	7, 5.9%			<0.05	<0.05	>0.05
Theft	113, 40.4%	1, 3.1%	41, 31.8%	71, 59.7%			<0.05	<0.05	<0.05
Fraud	14, 5.0%	0, 0.0%	8, 6.2%	6, 5.0%			−	−	−
Drug-pusher	20, 7.1%	0, 0.0%	10, 7.8%	10, 8.4%			−	−	−
Violence crime (*n*, %)^b^	133, 47.5%	31, 96.9%	70, 54.3%	32, 26.9%	59.024	<0.001	<0.05	<0.05	<0.05
Money or property as the aim (*n*, %)	147, 52.5%	1, 3.1%	59, 45.7%	87, 73.1%	53.918	<0.001	<0.05	<0.05	<0.05
Recidivism (*n*, %)	112, 40.0%	21, 65.6%	76, 58.9%	15, 12.6%	65.197	<0.001	>0.05	<0.05	<0.05

IED, intermittent explosive disorder; PC, psychopathology controls, HC; healthy controls.

a Chinese RMB (yuan) minimum monthly salary (per person).

b Violence crime refers to homicide, rape, assault, robbery, and affray. Nonviolence crime refers to theft, fraud, and drug-pusher.

### Psychopathology

Among the juvenile delinquents, the most frequent mental disorder was CD (*n* = 111). In addition, ADHD (*n* = 54), ODD (*n* = 31), SUD (*n* = 30), and AD (*n* = 7) were also found. No respondents were found to have affective disorder, mental retardation, or any other psychotic disorders at the time of assessment. The differences in psychopathology between the IED and PC groups are displayed in **Table 2**. Participants in the IED group had significantly less substance use disorders than participants in the PC group. No differences with other diagnoses were found between the two groups.

**Table 2:** Number of participants endorsing DSM-IV disorders.

	IED *n* = 32	PC *n* = 129	χ2	*P*
Anxiety disorder	1, 3.1%	6, 4.7%	0.144	1.000
Attention Deficit Hyperactivity Disorder	8, 25.0%	46, 37.4%	1.307	0.300
Conduct Disorder	17, 53.1%	84, 65.1%	1.577	0.226
Oppositional Defiance Disorder	7, 21.9%	24, 18.6%	0.176	0.627
Substance use disorder	1, 3.1%	29, 22.5%	6.336	0.010

DSM-IV, Diagnostic and Statistical Manual of Mental Disorders, Fourth Edition; IED, intermittent explosive disorder; PC, psychopathology controls.

### Anger and Anger Expression/Control

Compared with the HC group, the IED group displayed a higher level of state anger, trait anger, and anger expression-out (*P* < 0.05). The IED group also had a lower level of anger control-out than the PC and HC groups (*P* < 0.01). No difference on state anger, trait anger, anger control-in, and anger expression were found between the IED and PC groups (**Table 3**).

**Table 3:** Anger and aggressive behavior in different group.

	IED *n* = 32	PC *n* = 129	HC *n* = 119	*F*/χ2	*P*	IED vs. PC	IED vs. HC	PC vs. HC
**STAXI-2**								
State Anger	28.78 ± 7.68	30.06 ± 9.69	25.37 ± 7.49	9.408	<0.001	0.451	0.047	<0.001
Trait Anger	22.16 ± 4.86	22.32 ± 7.19	19.57 ± 5.92	6.082	0.003	0.899	0.045	0.001
Anger Control-In	18.88 ± 5.99	19.52 ± 5.67	19.61 ± 5.62	0.217	0.805	−	−	−
Anger Control-Out	15.31 ± 5.02	18.68 ± 6.03	18.89 ± 6.07	4.873	0.008	0.004	0.003	0.783
Anger Expression-In	15.63 ± 4.47	17.63 ± 5.33	16.52 ± 4.97	2.653	0.072	−	−	−
Anger Expression-Out	18.06 ± 4.05	17.81 ± 6.20	15.87 ± 5.13	4.485	0.012	0.821	0.048	0.006
**MOAS**								
Verbal Aggression	1.94 ± 1.13	1.28 ± 1.27	0.74 ± 1.25	31.335	<0.001	0.021	<0.001	<0.001
Against Property	1.30 ± 1.16	0.55 ± 0.93	0.24 ± 0.56	37.242	<0.001	<0.001	<0.001	0.019
Auto aggression	0.41 ± 0.95	0.18 ± 0.58	0.17 ± 0.63	4.433	0.109	−	−	−
Physical Aggression	1.70 ± 1.05	1.43 ± 1.41	0.48 ± 0.96	44.503	<0.001	0.433	<0.001	<0.001
Total score (weighted)	15.56 ± 5.14	8.26 ± 7.15	3.43 ± 5.41	69.669	<0.001	<0.001	<0.001	<0.001

STAXI-2, State-Trait Anger Expression Inventory; IED, intermittent explosive disorder; PC, psychopathology controls; HC, healthy controls.

### Aggressive Behaviors

During the first month in juvenile reformatory, the IED group showed not only a higher MOAS weighted total score (*P* < 0.001) but also higher verbal and property aggression scores compared to the PC and HC groups (*P* < 0.001). In physical aggression, the IED group’s level was higher than the HC group, but there was no difference with the PC group. No statistically significant difference in auto aggression was found between the three groups (**Table 3**).

## Discussion

We presented the first study to investigate the phenomenon of IED in male juvenile offenders in Shanghai, the most populous city in China. Some findings in our research may contribute to the understanding of the linkage between IED and juvenile delinquency in China’s urban areas.

Using the DSM-IV criterion, previous studies had found a comparatively high prevalence of IED in metropolitan China, namely, Shen’s 1.7% in Beijing and Shanghai (14) and Duan’s 3.39% in Shenzhen (24). Our finding of 11.4% participants meeting the criteria of IED cannot be directly compared with these studies as this was not an epidemiological study. Also, we used the IED research criteria that was broader than the DSM-IV IED criteria and closer to the definition of the Diagnostic and Statistical Manual of Mental Disorders, Fifth Edition (DSM-5) (19). That said, it clearly demonstrates that the prevalence of IED in juvenile offenders cannot be ignored.

Further, research has showed that a considerable number of juvenile crime and delinquency cases in urban China were committed by adolescents who had migrated from rural areas (25). We found this phenomenon exists not only in male juvenile offenders with no DSM-IV diagnosis (58.8%), but also in the IED group (50%) and other DSM-IV diagnosis groups (66.7%). This confirms the seriousness of juvenile delinquency among the migrant population in China. Meanwhile, it also showed that IED is a problem that cannot be ignored in both juvenile offender subgroups, urban and migrants.

Individuals with IED, including adolescents, usually had significantly more major lifetime non-personality disorders, including mood disorder, anxiety, and substance dependence (7, 26–30). We also found that a considerable proportion of IED juvenile offenders were comorbid with other DSM-IV disorders, such as conduct disorder, attention deficit hyperactivity disorder, and oppositional defiance disorder; however, the proportion was not statistically different when compared to the PC group.

We did not find any mood disorders in IED participants. In fact, we did not find any participants who met the diagnostic criteria of affective disorder, mental retardation, or any other psychotic disorders during the study period. This is because offenders suffering from these disorders are usually judged as “excused,” having “extenuated criminal responsibility,” or having “no ability to serve a sentence” and thus placed in other institutions. Although depressive disorders could be secondary to their incarceration, our interviews were conducted with offenders who had been in the reformatory no more than thirty days, thus making it difficult to identify depressive episodes within the group. Without doubt, these interviewees may have had affective disorders or other psychotic disorders in the past. However, this study focused primarily on their mental status during their imprisonment. Meanwhile, the IED participants had no significant differences with the other two groups in terms of age and education levels. Thus, age, education level, and psychopathology were not included as covariates in the final analyses.

We examined levels of anger and aggression in three groups of male juvenile offenders. Although the limited sample size (32 IED participants) might lead to certain negative results, we found some difference on STAXI-2 and MOAS scores between the IED and two control groups. Compared with the HC group, the IED group showed a significantly higher anger trait as well as deficiencies in anger control-out ability. This made them more susceptible to experiencing bouts of spontaneous anger; therefore, they also reported higher state anger and anger expression-out. These findings are consistent with previous research findings where IED groups showed increased anger (31).

Studies have shown increased anger in individuals with IED relative to psychopathology control groups (26, 32) and non-aggressive personality disorder groups (33). In this research, the IED and PC groups reported similar levels of anger experience and expression except in the case of anger control ability. The lack of significant difference between offenders with IED and psychopathology control groups may be attributed to the existence of comorbidity in the IED group. The high rate of comorbidity diagnoses might explain the features found in both the IED and psychopathology control groups (26). Further, the similarities between the two groups may have resulted from the diagnostic composition of the PC group in our research. Compared to previous studies that had significant percentages of mood disorder in their PC groups (7, 26–30), our PC group was mainly comprised of those with various externalized disorders, such as ADHD, ODD, and CD, all of which were also characterized by high levels of anger experience and expression (34, 35).

Despite the similarities in anger experience between the IED and PC groups, the IED group showed higher verbal and property aggression compared to the PC and HC groups. Although physical aggression levels in the IED group were similar to the PC group, after weighting all items, the total aggression level of the IED group was the highest of all three groups. The high aggression of IED offenders in the juvenile reformatory poses a considerable risk to staff as well as to non- or less-violent prisoners. Therefore, they should be supervised with additional caution.

We hypothesized that the criminal profiles of juvenile offenders with IED would differ from those of other juvenile offenders. As predicted, participants in the IED group were more likely to be repeat offenders and perpetrators of violent crimes. These results are consistent with DeLisi’s previous findings that federal correctional clients with IED had a dramatically higher correlation with recidivism, total arrest charges, and assault-related charges (5). For individuals diagnosed with IED, their aggressive behavior was not usually intended to achieve some tangible objective like money, power, or intimidation (1). We also found that they were less prone to state money as their primary motive for crime. This might be explained by the high rate of reactive aggression and the low rate of premeditated aggression among IED subjects (36). Thus, they were more likely to be involved in violent crimes, such as rape, assault, and affray rather than planned antisocial behavior related to money, such as robbery and theft. This tendency is confirmed by the lower rate of IED participants attributing money as their motives for crime in their self-reports. This implies a discomforting situation regarding juvenile delinquents in the IED group, that is, the differences suggest the need to develop individualized crime prevention strategies for at-risk IED groups.

When interpreting our results, certain limitations should be considered. First, as a cross-sectional study, the ability to address the causality of the factors was limited. Therefore, further longitudinal studies are needed. Second, when answering questions, respondents may have tended to adhere to social norms rather than actual situations. That said, as the three groups of juvenile offenders were from the same correctional institution, there was no reason to suspect that one of the groups had more obvious misreporting issues when answering these questions, and the comparisons are probably valid. Third, the study only examined the situation of IED among male juvenile offenders in one reformatory in Shanghai. We may not be able to generalize these results to serve as indicators for female IED juvenile offenders or IED adolescents without criminal records in other cities of China. Finally, given the prevalence of depression in prisons in other countries (3, 5, 6), this may also impact the generalizability of the findings. Therefore, further research related to IED must be conducted across a broader population.

Despite these limitations, the study contributes to our understanding of IED in juvenile offenders in China. This is a problem that cannot be ignored by policymakers or mental health and criminal justice professionals. The relationship between IED with excessive anger and aggression implies that specific and individually tailored intervention approaches should be developed to correct IED juvenile offenders’ behavior in order to help prevent further crimes. Research in the near future should focus on the risk and protective factors of the disease, the creation of feasible screening and early detection strategies, and the development of effective treatment methods Manuscript Formatting.

## Ethics Statement

This study was carried out in accordance with the recommendations of Research Ethical Committees at Shanghai Mental Health Center with written informed consent from all subjects. All subjects gave written informed consent in accordance with the Declaration of Helsinki. The protocol was approved by the Research Ethical Committees at Shanghai Mental Health Center (IORG Number: IORG0002202; FWA Number: FWA00003065).

## Author Contributions

YS and BX contributed to the conception of the study. YS, YQ, and MZ performed the investigation. YS and YQ contributed equally to analysis and manuscript preparation. BX reviewed and approved the final version of manuscript.

## Funding

This work was supported by grants from the National Natural Science Foundation of China (81302624, PI: YQ; 81202388, PI: YS), the Shanghai three-year action plan of public health (fourth round)-senior overseas research team training program (GWTD2015509, PI: BX), and the Shanghai Municipal Health and Family Planning Commission (psychiatry) key discipline (2017ZZ02021, PI: MZ).

## Conflict of Interest Statement

The authors declare that the research was conducted in the absence of any commercial or financial relationships that could be construed as a potential conflict of interest.
